# Moiré pattern of interference dislocations in condensate of indirect excitons

**DOI:** 10.1038/s41467-021-21353-7

**Published:** 2021-02-19

**Authors:** J. R. Leonard, Lunhui Hu, A. A. High, A. T. Hammack, Congjun Wu, L. V. Butov, K. L. Campman, A. C. Gossard

**Affiliations:** 1 Department of Physics, University of California at San Diego, La Jolla, CA USA; 2 Materials Department, University of California at Santa Barbara, Santa Barbara, CA USA

**Keywords:** Bose-Einstein condensates, Two-dimensional materials

## Abstract

Interference patterns provide direct measurement of coherent propagation of matter waves in quantum systems. Superfluidity in Bose–Einstein condensates of excitons can enable long-range ballistic exciton propagation and can lead to emerging long-scale interference patterns. Indirect excitons (IXs) are formed by electrons and holes in separated layers. The theory predicts that the reduced IX recombination enables IX superfluid propagation over macroscopic distances. Here, we present dislocation-like phase singularities in interference patterns produced by condensate of IXs. We analyze how exciton vortices and skyrmions should appear in the interference experiments and show that the observed interference dislocations are not associated with these phase defects. We show that the observed interference dislocations originate from the moiré effect in combined interference patterns of propagating condensate matter waves. The interference dislocations are formed by the IX matter waves ballistically propagating over macroscopic distances. The long-range ballistic IX propagation is the evidence for IX condensate superfluidity.

## Abstract (web summary)

Previous interference experiments on indirect excitons found dislocation-like phase singularities that could not be explained by common phase defects. Here, the authors explain these features in terms of the moiré pattern of interference of condensate matter waves propagating over macroscopic distances.

## Introduction

Phase defects in quantum states play a significant role both in the state properties and in the transition between the states. For instance, a transition to 2D superfluid state is governed by pairing of vortices and, in turn, unpaired vortices can cause dissipations for particle fluxes. Phase defects are studied in quantum states of matter ranging from superconductors and superfluid Helium^1^ to condensates of atoms^2–9^, magnons^10^, polaritons^11–20^, and excitons^7,8,20–29^. A variety of phase defects is considered, including vortices^2–4,10,12,14^, polarization vortices^21,24,25^, half-vortices^1,7,8,11,13,27^, skyrmions^5,7,8,19,26,28^, solitons^6,15–19^, striped phases^9,22,23,25–27^, and phase domains^29^. Vortices and other phase defects can be revealed by characteristic features in interference patterns produced by the quantum system.

**Fig. 1:**
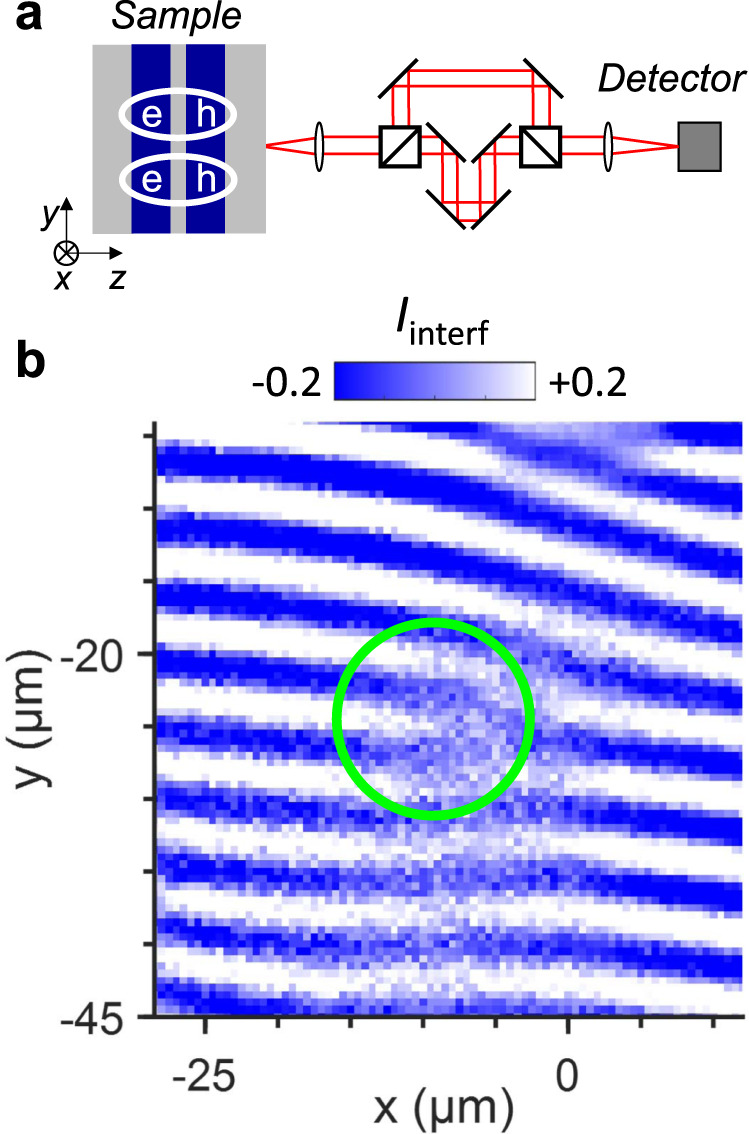
Dislocation-like singularity in the exciton interference pattern. **a** CQW diagram. AlGaAs (gray) and GaAs (blue). Ovals indicate IXs composed of electrons (e) and holes (h). Diagram of the interferometric setup is shown on the right. The emission images produced by each of the two arms of the Mach–Zehnder interferometer are shifted with respect to each other in the *x*–*y* plane. **b** Measured interference pattern ${I}_{{\rm{interf}}}(x,y)$. The isolated interference dislocation is marked by a green circle.

Quantum states of excitons can be created in a system of indirect excitons (IXs), aka interlayer excitons^30^. IXs are formed by electrons and holes in spatially separated layers in coupled quantum well (CQW) heterostructures (Fig. 1a). Due to the electron–hole separation, IXs have long lifetimes within which they can cool below the temperature of quantum degeneracy and form a condensate in momentum space^30^. The IX condensation is detected by the measurement of IX spontaneous coherence with a coherence length much larger than in a classical gas^21^.

Excitons can transform to photons that gives an opportunity to probe exciton matter waves by measuring optical interference patterns. Superfluidity in Bose–Einstein condensates of excitons can enable long-range ballistic exciton propagation and can lead to emerging nontrivial long-scale interference patterns. However, the pioneering theoretical works on exciton superfluidity indicated its suppression by interband transitions, that is by electron–hole recombination for semiconductor systems and tunneling for semimetal systems^31,32^. The theory predicts that the separation between the electron and hole in IX reduces the recombination rate by orders of magnitude thus enabling IX superfluid propagation over macroscopic distances^30^.

In this work, we present dislocation-like singularities in interference patterns in the condensate of IXs. We analyze how exciton vortices, polarization vortices, half-vortices, skyrmions, and half-skyrmions should appear in the interference experiments, and show that the observed dislocations in interference patterns are not associated with any of these known phase defects. We present the origin of these singularities in condensate interference patterns: The observed interference dislocations originate from the moiré effect in the combined interference patterns of ballistically propagating condensate matter waves. The ballistic IX propagation over macroscopic distances is the evidence for IX condensate superfluidity.

## Results

A cold gas of IXs is realized in regions of external ring and localized bright spot (LBS) rings in IX emission. These rings form on the boundaries of electron-rich and hole-rich regions created by current through the structure and optical excitation, respectively (ref. ^33^ and references therein). LBS sources are stable and well-defined sources of cold IXs^33^, we use them here for exploring phenomena in propagating condensate matter waves.

Figure 1b shows the interference pattern of IX emission measured by shift-interferometry. This method is outlined below. As a result of IX recombination, IXs transform to photons. These photons go through the Mach–Zehnder interferometer (Fig. 1a). Each of the two interferometer arms forms an image of the IX emission on CCD detector. These two images are shifted relative to each other in *x*-direction so that the measured interference pattern is produced by the interference between the emission of IXs separated by the shift *δx* in CQW plane. The measurements were performed at temperatures *T* ≥ 100 mK in an optical dilution refrigerator. Details are described in the “Methods” section.

Figure 1b shows a dislocation-like phase singularity in the interference pattern. The origin of the interference dislocations is uncovered below.

We start from considering exciton vortices and skyrmions. Dislocations (forks) in interference patterns can be associated with vortices in quantum systems. In a singly quantized vortex, the phase of the condensate winds by 2*π* around the singularity point that can be revealed as a fork-like defect in an interference pattern. Forks in interference patterns were reported for vortices in atom condensates^3^, polariton vortices^12–14^, magnon vortices^10^, and optical vortices^34^.

**Fig. 2:**
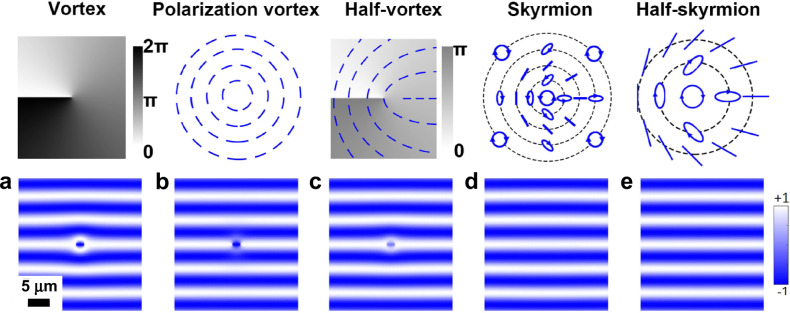
Simulated exciton interference patterns for vortices and skyrmions. Interference patterns ${I}_{{\rm{interf}}}(x,y)$ for an exciton phase vortex (**a**), polarization vortex (**b**), half-vortex (**c**), skyrmion (**d**), and half-skyrmion (**e**) for the shift-interferometry corresponding to the experiment (Fig. 1). The phase and polarization patterns are shown on the top: the phase is presented by the color and the linear, circular, and elliptic polarizations are presented by bars, circles, and ellipses, respectively. These simulated interference patterns are drastically different from the experiment (Fig. 1b), indicating that the observed interference dislocation (Fig. 1b) is not associated with a vortex, or polarization vortex, or half-vortex, or skyrmion, or half-skyrmion.

However, a vortex can lead to the appearance of dislocation in an interference pattern only for certain interferometric experiments, in particular for the interference of a vortex field with a plane wave. For the shift-interferometry experiments, simulations show that a vortex should lead to the appearance of a pair of left- and right-oriented dislocations of interference fringes separated by a distance equal to the shift *δx* (Fig. 2a). The interference pattern for a vortex (Fig. 2a) is drastically different from the isolated interference dislocation, which is separated from other dislocations by distances much larger than the shift *δx*, in the experiment (Fig. 1b). This indicates that the observed interference dislocation is not associated with a vortex.

We also simulated the interference patterns for exciton polarization vortices, half-vortices, skyrmions, and half-skyrmions (Fig. 2b–e). In contrast to vortices (Fig. 2a–c), no pair of left- and right-oriented interference dislocations separated by the shift *δx* is observed for skyrmions (Fig. 2d, e) that reflects the lack of singular phase winding for the latter. These interference patterns are also drastically different from the isolated interference dislocation in the experiment (Fig. 1b). This indicates that the observed interference dislocation is not associated with a polarization vortex, or half-vortex, or skyrmion, or half-skyrmion.

The simulations provide a guide for identifying phase defects in interferometric experiments. The simulations show that the existence of vortices should lead to the appearance of pairs of left- and right-oriented interference dislocations separated by the shift *δx* (Fig. 2a–c) and that skyrmions are not revealed in the interference pattern (Fig. 2d, e). (Additional polarization measurements are needed for distinguishing different vortices in Fig. 2a–c and for revealing skyrmions, this will be considered elsewhere.) In contrast to the vortex or skyrmion interference patterns, the experiment (Fig. 1b) detects an isolated interference dislocation. This shows that the observed interference dislocation is a phenomenon, different from known phase defects such as vortices and skyrmions.

The origin of the observed interference dislocations is outlined below. In the shift-interferometry experiment, IXs with wavefunction *ψ*(**r**) produce the interference pattern $I({\bf{r}})=| \psi ({\bf{r}}- \delta {\bf{r}}/2){e}^{i{q}_{t}y}+\psi ({\bf{r}}+\delta {\bf{r}}/2){| }^{2}$, where *q*_*t*_ = 2*πα*/*λ* sets the period of the interference fringes, *α* is a small tilt angle between the image planes of the interferometer arms, and *λ* is the emission wavelength.

**Fig. 3:**
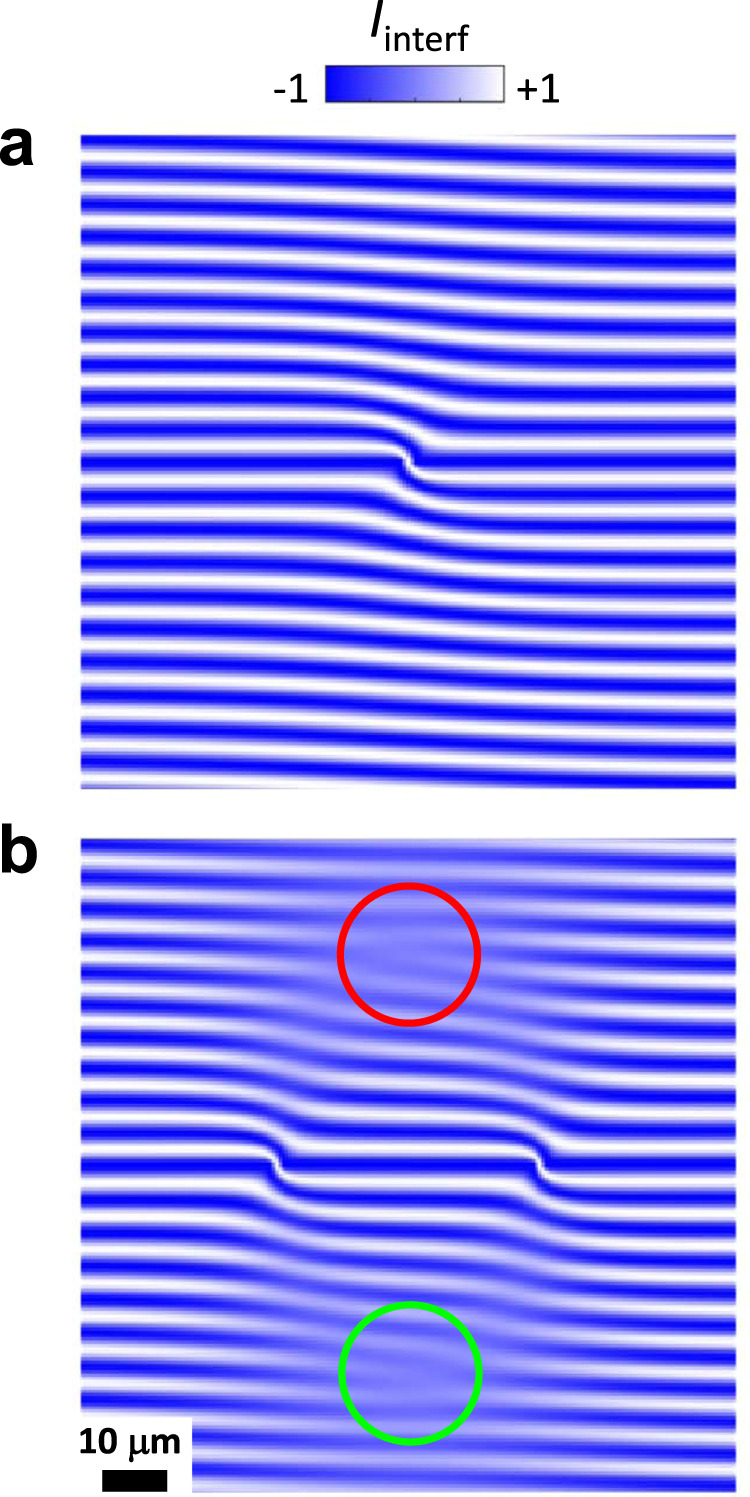
Simulated exciton interference pattern with interference dislocations. **a** Shift-interference pattern ${I}_{{\rm{interf}}}(x,y)$ for a radial IX condensate matter wave propagating from a source in the center. *k* = 1.5 μm^−1^. **b** ${I}_{{\rm{interf}}}(x,y)$ for two radial IX condensate matter waves propagating from two sources separated along **x**. The interference dislocations (marked by green and red circles) are observed at the locations determined by angle $\gamma =\arccos [{(k\delta x)}^{-1}\pi /2]$ as described in the text. *γ* is the angle between the line connecting the sources and the direction from the source to the interference dislocation. The interference dislocations originate from the moiré effect in the combined interference patterns of radial IX condensate matter waves propagating from two sources.

First, we consider a single source of IXs. IX condensate produced by a source is described by a single macroscopic wavefunction approximated by a radial IX matter wave with *ψ*(**r**) ∝ *e*^*ikR*^, where *R* = ∣**r** − **r**_s_∣ is the distance to the source, **r**_s_ the source location, and **k** = *k*(**r** − **r**_s_)/*R* the IX momentum. (In comparison, uncondensed IXs produced by a source are described by many different matter waves with a wide spread of momenta and short scattering times. Estimates for the IX scattering times are presented in Supplementary Note 3.) For the shift *δ***r** = *δ***x**, the interference pattern is given by $I(x,y) \sim \cos ({\bf{k}}\delta {\bf{x}}+{q}_{t}y)$ shown in Fig. 3a.

Now, we consider two radial IX condensate matter waves produced by two IX sources. For simplicity, to outline the origin of the phase singularities, we consider the waves of equal strength and with equal *k* (Fig. 3b). For the two IX condensate matter waves, the interference pattern is given by

1 $$I(x,y) \sim \frac{1}{{R}_{1}}\cos ({{\bf{k}}}_{1}\delta {\bf{x}}+{q}_{t}y)+\frac{1}{{R}_{2}}\cos ({{\bf{k}}}_{2}\delta {\bf{x}}+{q}_{t}y),$$

where *R*_*i*_ = ∣**r** − **r**_s,*i*_∣ and **k**_*i*_ = *k*(**r** − **r**_s,*i*_)/*R*_*i*_ are the distance to the source and momentum for IXs propagating from source *i* with *i* = 1, 2. In this expression, each source generates a coherent IX condensate matter wave propagating ballistically from the source with no dissipation and *I*(*x*, *y*) intensities produced by source 1 and source 2 are added. The addition of intensities corresponds to the lack of coherence between the IX matter waves propagating from source 1 and source 2 and is in accord with the experiment: the IX creation from optically generated holes and electrically generated electrons in LBS sources^33^ suggests the lack of coherence between different LBS sources.

The simulations of converging IX condensate matter waves (Eq. (1), Fig. 3b) reproduce the isolated dislocations observed in the experiment (Fig. 1b). Their origin is outlined below. The interference dislocations (Fig. 3b) originate from the moiré effect in the combined interference patterns of propagating condensate matter waves. The radial condensate matter waves propagating from source 1 and from source 2 create similar interference patterns (Fig. 3a) shifted relative to each other by the separation between the sources **r**_s,1_ − **r**_s,2_. Combining these two patterns is presented by Eq. (1). [Factors 1/*R*_*i*_ in Eq. (1) correspond to 2D radial condensate matter waves propagating without dissipation from their sources. According to the definition of interference patterns (see “Methods”), this factor is not essential for the single-source pattern, factors 1/*R*_*i*_ appear for two sources for weighting their contributions.] This combining of separated interference patterns forms a moiré pattern. To visualize this moiré effect, we present Supplementary Movies 1 and 2 showing how the interference dislocations appear and how their locations change when the two combining patterns of interference fringes move relative to each other.

The locations of the interference dislocations originating from the moiré effect are given by the ballistic propagation of IXs. For two radial IX condensate matter waves produced by two sources separated along the shift *δ***x**, on the bisector line, Eq. (1) gives $I \sim \cos ({q}_{t}y)\cos (k\delta x\cos \gamma )$, where *γ* is the angle between **r** − **r**_s1_ and *δ***x**. In this equation, the first factor oscillates along *y*, and the second factor produces the beating pattern. When $k\delta x\cos \gamma =\left(n+\frac{1}{2}\right)\pi$ with *n* an integer, the second factor changes sign and this phase slip creates the phase singularity in interference pattern. Numerical simulations (Fig. 3b) confirm that the dislocations in interference pattern are observed at the locations determined by this equation. The bifurcation of the interference pattern at the phase slipping point is described in Supplementary Note 1.

**Fig. 4:**
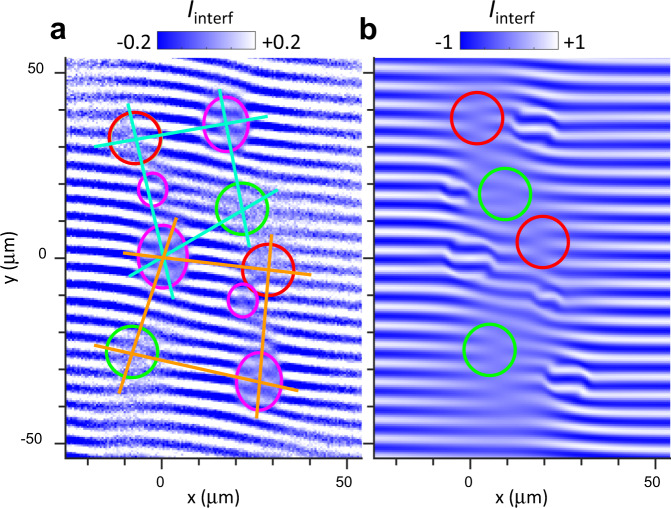
Measured and simulated moiré patterns of interference dislocations. **a** Measured shift-interference patterns ${I}_{{\rm{interf}}}(x,y)$ for IXs in the region of five LBS sources. Three strong sources are clearly seen due to the pronounced phase domains at the source locations. These phase domains are associated with the Pancharatnam–Berry phase and are described in ref. ^29^. Two weaker sources are also seen ~20 μm above the bottom and medium strong sources. Sharp phase shifts of interference fringes embracing a phase domain of interference fringes in a circular region around each LBS source of IXs are marked by magenta lines. The right- and left-oriented dislocations in the interference pattern are marked by red and green circles, respectively. All sources participate in the formation of these dislocations. A stronger contribution to the upper (lower) pair of right- and left-oriented dislocations is given by the two upper (lower) strong sources. This is illustrated by cyan (orange) lines between the dislocations and the sources giving stronger contribution. These lines form diamond shapes similar to the diamond shapes formed by the lines connecting interference dislocations and two sources producing them in Fig. 3 and Supplementary Fig. 1. **b** Simulated ${I}_{{\rm{interf}}}(x,y)$ for five radial IX condensate matter waves ballistically propagating from five sources positioned at the locations of LBS sources. The phase domains described in ref. ^29^ are also shown. The simulations (**b**) qualitatively reproduce the interference dislocations marked by red and green circles in the experiment (**a**).

Details of the experiment are presented next. In the experimental system, there are several LBS sources of IXs (Fig. 4a). Three strong sources are clearly seen due to the pronounced phase domains at the source locations. Two weaker sources are also seen in Fig. 4a. Sharp phase shifts of interference fringes embracing a phase domain of interference fringes in a circular region around each LBS source of IXs are marked by magenta lines. These sharp phase shifts and, in turn, the phase domains are associated with the Pancharatnam–Berry phase and are described in ref. ^29^ and outlined in Supplementary Note 5. The right- and left-oriented interference dislocations are marked by red and green circles. All sources participate in the formation of these dislocations. A stronger contribution to the upper (lower) pair of right- and left-oriented dislocations is given by the two upper (lower) strong sources. This is illustrated by cyan (orange) lines between the dislocations and the sources giving stronger contribution. These lines form diamond shapes similar to the diamond shapes formed by the lines connecting interference dislocations and two sources producing them in Fig. 3 and Supplementary Fig. 1.

Simulated interference pattern for five radial IX condensate matter waves propagating from five sources with $I(x,y) \sim \mathop{\sum }\nolimits_{i = 1}^{5}{P}_{i}/{R}_{i}\cos ({{\bf{k}}}_{i}\delta {\bf{x}}+{q}_{t}y)$ is presented in Fig. 4b. This equation is a five-source analog of Eq. (1), *R*_*i*_ and **k**_*i*_ are the distance to the source and momentum for IXs propagating from source *i*. The values of *k* are estimated independently of the analysis of interference dislocations, namely, from the position of interference fringes in the source vicinity including the sharp phase shifts of interference fringes around the source as described in ref. ^29^. The source powers *P*_*i*_ are estimated from the IX emission intensities in the source region. The sources in the simulations are positioned at the locations of LBS sources in the experiment. The simulations (Fig. 4b) qualitatively reproduce the interference dislocations marked by red and green circles observed in the experiment (Fig. 4a).

The interference dislocations are formed by the IX condensate matter waves ballistically propagating from the IX sources to the locations of interference dislocations over distances reaching 30 microns. This ballistic IX propagation described by the multisource analog of Eq. (1) produces the interference pattern shown in Fig. 4. The long-range ballistic IX propagation over these macroscopic distances shows that IXs propagate without scattering over dramatically long times, orders of magnitude longer than in classical exciton gas (estimates for the IX scattering times are presented in Supplementary Note 3), and, therefore, is the evidence for IX condensate superfluidity.

All observed phase singularities disappear above the IX condensation temperature where the interference patterns become trivial with continuous interference fringes showing no dislocations (Supplementary Fig. 2a and Supplementary Note 6). This is consistent with the requirement of long-range ballistic IX propagation, which is provided by the condensate superfluidity.

As outlined above, in earlier studies of quantum systems, the mechanisms, which can lead to the appearance of dislocations (forks) in interference patterns were based on vortices. Our work introduces another mechanism, which can lead to the appearance of dislocations—the moiré effect. The moiré effect should be considered in analyses of interference patterns and particularly in identifying the origin of dislocations in interference patterns.

In summary, we present dislocation-like singularities in interference patterns in condensate of indirect excitons. We show how various exciton vortices and skyrmions should appear in the interference experiments. The observed dislocations in interference patterns are not associated with any of these known phase defects. We present the origin of these singularities in condensate interference patterns: The observed interference dislocations originate from the moiré effect in the combined interference patterns of propagating condensate matter waves. The moiré effect is a mechanism, which leads to the appearance of dislocations in interference patterns. These results can be used for identifying vortices, skyrmions, and moiré effects in interference patterns of quantum systems. The interference dislocations are formed by the IX matter waves ballistically propagating over macroscopic distances. The long-range ballistic IX propagation is the evidence for IX condensate superfluidity.

## Methods

### Coupled quantum well heterostructure

The *n* − *i* − *n* GaAs/AlGaAs CQW heterostructure was grown by molecular beam epitaxy. The *i* region consists of a single pair of 8-nm GaAs QWs separated by a 4-nm Al_0.33_Ga_0.67_As barrier and surrounded by 200-nm Al_0.33_Ga_0.67_As layers. The *n* layers are Si-doped GaAs with Si concentration 5 × 10^17^ cm^−3^. The indirect regime where IXs form the ground state is realized by the voltage applied between *n* layers. The small in-plane disorder in the CQW is indicated by the emission linewidth of 1 meV. IXs cool to temperatures within ~50 mK of the lattice temperature^35^, which was lowered to 100 mK in an optical dilution refrigerator. This cools IXs well below the temperature of quantum degeneracy, which is in the range of a few kelvin for typical IX density 10^10^ cm^−2 ^^35^.

### Experimental setup

The photoexcitation is provided by a 633-nm HeNe laser, more than 400 meV above the energy of IXs and farther than 80 μm away from the studied region, IX coherence is not induced by photoexcitation and forms spontaneously. LBS are sources of cold IXs due to their separation from the laser excitation spot. The emission is split between the arms of Mach–Zehnder (MZ) interferometer (Fig. 1). The path lengths of the arms are equal. After the interferometer, the emission is filtered by an interference filter of linewidth ±5 nm adjusted to the IX emission wavelength *λ* ~ 800 nm. This filter rejects the emission of both spatially direct excitons and bulk GaAs, which is spectrally separated from the IX emission, but does not affect the IX emission, since the filter linewidth is much larger than the IX linewidth. Each of the two interferometer arms forms an image of IX emission on a liquid-nitrogen cooled CCD detector. The interfering emission images produced by arm 1 and arm 2 of the MZ interferometer are shifted relative to each other along *x*-direction to measure the interference between the emission of IXs, which are separated by *δx* in the CQW plane. The interference pattern is given by ${I}_{{\rm{interf}}}=(I-{I}_{1}-{I}_{2})/(2\sqrt{{I}_{1}{I}_{2}})$, where *I*_1_ is IX emission intensity for arm 1 open, *I*_2_ for arm 2 open, and *I* for both arms open. A small tilt angle *α* between the image planes of the two arms introduces a phase difference component linear in *y*, the coordinate in the direction perpendicular to the tilt axis, which produces periodic oscillation of ${I}_{{\rm{interf}}}$. The period of the interference fringes is set by *q*_*t*_ = 2*πα*/*λ*. The shift in the shift-interferometry experiments *δx* = 2 μm. This shift is suitable for these experiments because it is smaller than the coherence length, smaller than the characteristic sizes of the features in the interference patterns, and larger than the 1.5 μm spatial resolution in the experiment. The simulations are performed for the same shift.

## Supplementary information

Supplementary Information

Description of Additional Supplementary Files

Supplementary Movie 1

Supplementary Movie 2

## Data Availability

All relevant data are available from the authors.
