# Supplementary material for: Moiré pattern of interference dislocations in condensate of indirect excitons
Source: Nat Commun. 2021 Feb 19;12:1175. doi: 10.1038/s41467-021-21353-7 (PMC7895953; doi:10.1038/s41467-021-21353-7)
Supplement: Supplementary file 1 — Supplementary Information [file 41467_2021_21353_MOESM1_ESM.pdf]

Supplementary information: Moiré pattern of interference dislocations in condensate of indirect excitons

J. R. Leonard,[1] Lunhui Hu,[1] A. A. High,[1] A. T. Hammack,[1]
Congjun Wu,[1] L. V. Butov,[1] K. L. Campman,[2] and A. C. Gossard[2]

[1]*Department of Physics, University of California at San Diego, La Jolla, California 92093-0319, USA*
[2]*Materials Department, University of California at Santa Barbara, Santa Barbara, California 93106-5050, USA*

Supplementary Note 1: The bifurcation of the interference pattern at the phase slipping point

As outlined in the main text, the dislocations in interference pattern are observed at the phase slipping locations determined by equation $k\delta x \cos\gamma = \left(n + \frac{1}{2}\right)\pi$ with n an integer. For the region close to the bisector line, i.e. for $x'/R \ll 1$ with x' the distance of the location (x, y) to the bisector and $R = (R_1 + R_2)/2$, Eq. (1) in the main text gives

$$I \sim \frac{2}{R}\cos\left(q_t y + \frac{kx'}{R}\sin^2\gamma\right)\cos\left(k\delta x\cos\gamma\right) + \frac{2x'\cos\gamma}{R^2}\sin q_t y \sin\left(k\delta x\cos\gamma\right) + I_{bg}\,, \tag{1}$$

where I_{bg} is the background. If we move slightly leftward ($x' < 0$) or rightward ($x' > 0$) away from the phase slipping point on the bisector line, the 2nd term in Supplementary Eq. (1) dominates leading to the bifurcation of the interference pattern.

Supplementary Note 2: Interference dislocations for nonzero β

In Fig. 3b and related modeling in the main text, we considered the interference dislocations for two radial IX matter waves propagating from two IX sources separated along **x**. In this section, we extend this consideration to the case of nonzero angle β between the line connecting two sources of radial IX matter waves and **x**. In this case, the phase slip along the bisector line appears at angle γ given by $k\delta x\cos\gamma\cos\beta = \left(n + \frac{1}{2}\right)\pi$, where γ is the angle between the line connecting the sources and the direction from the source to the interference dislocation (Supplementary Fig. 1b). Numerical simulations (Supplementary Fig. 1a) confirm that the dislocations in interference pattern are located at γ given by this equation.

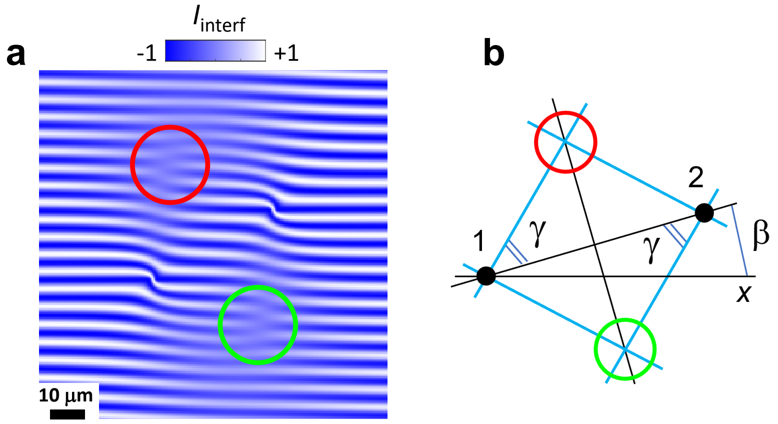

Supplementary Fig. 1. **Simulated exciton interference pattern with interference dislocations.** (a) Shift-interference patterns $I_{interf}(x, y)$ for two radial IX condensate matter waves propagating from two sources separated along the line at angle β relative to **x**. The interference dislocations (marked by green and red circles) are observed at the locations determined by angle $\gamma = \arccos\left[(k\delta x\cos\beta)^{-1}\pi/2\right]$. (b) Schematics showing sources of radial IX condensate matter waves (1 and 2), angles γ and β, and the interference dislocations (green and red circles).

Supplementary Note 3: On ballistic exciton propagation

Ballistic propagation over large distances with a large mean free path $l = v\tau$ can be achieved in a system with a large mean free time (scattering time) τ or in a system with a large velocity v. For instance, polaritons moving with large velocity $v_p \sim 10^8$ cm/s can ballistically propagate over large distances in the regime of fast scattering with τ in the picosecond range (e.g. for $v_p \sim 10^8$ cm/s, $l \sim 30\ \mu$m can be achieved with $\tau_p \sim 30$ ps).

The velocity of IX propagation $v_{IX} = \hbar k/m \sim 10^5$ cm/s is estimated from the IX momentum $k = 1.5\ \mu\text{m}^{-1}$ and IX mass $m = 0.22 m_0$ (m_0 is the mass of free electron). The IX mass is measured in Ref. [1], k is estimated from the interference pattern in the source vicinity using the method described in Ref. [2]. For the observed ballistic IX propagation over $l \sim 30\ \mu$m, the estimated IX scattering time $\tau_{IX} = l/v_{IX}$ exceeds 30 ns. This dramatically long scattering time achieved

in the IX condensate is the evidence for IX condensate superfluidity. In comparison, for a classical IX gas at 1.5 K with diffusion coefficient $D = 10$ cm^2/s in the CQW heterostructure, $\tau = mD/k_B T \sim 10$ ps [3].

Supplementary Note 4: A movie showing the moiré pattern of interference dislocations

To visualize the moiré effect in the combined interference patterns, we present a movie showing how the interference dislocations appear and how their locations change when the two combining patterns of interference fringes move relative to each other as described in the main text. In this movie, the bottom pattern of interference fringes is given by the first term in Eq. (1) in the main text and the top pattern of interference fringes is given by the second term in Eq. (1) in the main text. The top pattern is semitransparent so that the intensities of the bottom and top patterns add with the same weight in the overlapping region, visualizing Eq. (1) in the main text and showing the moiré pattern of interference dislocations. The overlapping region is marked by a dashed contour. We also present the movie with the interference dislocations marked by green and red circles.

Supplementary Note 5: The features of IX condensate interference patterns

For a classical exciton gas at high temperatures, the interference patterns are featureless (Supplementary Fig. 2a). However, for the IX condensate at low temperatures, the interference patterns are complex (Supplementary Fig. 2b). Different features of this complex interference patterns present different phenomena in IX condensate. These features are described in Refs. [4] and [2] ([21] and [29] in the main text) and in this work and are summarized in this section.

The first feature is a high amplitude of interference fringes, much higher than in a classical gas, at large shifts δx (large δx means δx larger than the thermal de Broglie wavelength λ_{dB} and the optical spatial resolution). This feature is addressed in Ref. [4] and is outlined in this paragraph. Coherence between IXs separated in the CQW plane by δx is quantified by the first order coherence function $g_1(\delta x)$. The measured amplitude of interference fringes $A_{interf}(\delta x)$ is given by the convolution of $g_1(\delta x)$ with the point-spread function (PSF) of the optical system [4]. The PSF width corresponds to the optical spatial resolution. For a classical IX gas, $g_1(\delta x)$ is narrow and $A_{interf}(\delta x)$ fits well to the PSF [4]. For the IX condensate, $g_1(\delta x)$ and, in turn, $A_{interf}(\delta x)$ extend to large δx, demonstrating spontaneous coherence [4]. Spontaneous coherence of IX matter waves is equivalent to condensation of IXs in momentum space referred to as Bose-Einstein condensation (BEC): the Fourier transform of $g_1(\delta x)$ gives the particle distribution in momentum space n_k and the width of $g_1(\delta x)$, the coherence length ξ, is inversely proportional to the width of n_k. In a classical gas, ξ is close to $\lambda_{dB} = (2\pi\hbar^2/mk_B T)^{1/2}$ and is small ($\xi_{classical} = \lambda_{dB}/\pi^{1/2} \sim 0.3$ μm at $T = 0.1$ K for IXs in GaAs CQW). The measurement of IX spontaneous coherence with $\xi \gg \xi_{classical}$ is a direct measurement of BEC of IXs (Fig. 3 in Ref. [4]).

The second feature is sharp phase shifts of interference fringes embracing a phase domain of interference fringes in a circular region around each LBS source of IXs. These sharp phase shifts are marked by magenta lines in Supplementary Fig. 2c. This feature is addressed in Ref. [2] and is outlined in this paragraph. Correlations are found between the sharp phase shifts, polarization pattern of IX emission, and onset of IX spontaneous coherence [2]. The correlation between the sharp phase shifts and the polarization changes shows that the sharp phase shifts originate from the Pancharatnam-Berry phase acquired in the condensate of IXs [2].

The third feature is isolated dislocations (forks) in the interference patterns. These dislocations, marked by green and red circles in Supplementary Fig. 2c, are separated from the sources of IXs by macroscopic distances reaching 30 microns. This feature is addressed in this work and is outlined in this paragraph. The interference dislocations originate from the moiré effect in the combined interference patterns of propagating IX condensate matter waves. The interference dislocations are formed by the IX matter waves ballistically propagating from the IX sources to the locations of interference dislocations over distances reaching 30 microns. This ballistic IX propagation described by the multisource analogue of Eq. (1) in the main text produces the interference pattern shown in Fig. 4. The long-range ballistic IX propagation over these macroscopic distances shows that IXs propagate without scattering over dramatically long times, orders of magnitude longer than in classical exciton gas (as outlined in Supplementary Note 3), and, therefore, is the evidence for IX condensate superfluidity.

There are relations between these distinctly different features of the IX condensate interference patterns. The relation between the first and second features: The locations of the sharp phase shifts correlate not only with the polarization change but also with the sharp enhancement of the amplitude of the interference fringes indicating that the Pancharatnam-Berry phase is acquired in the condensate of IXs as described in Ref. [2].

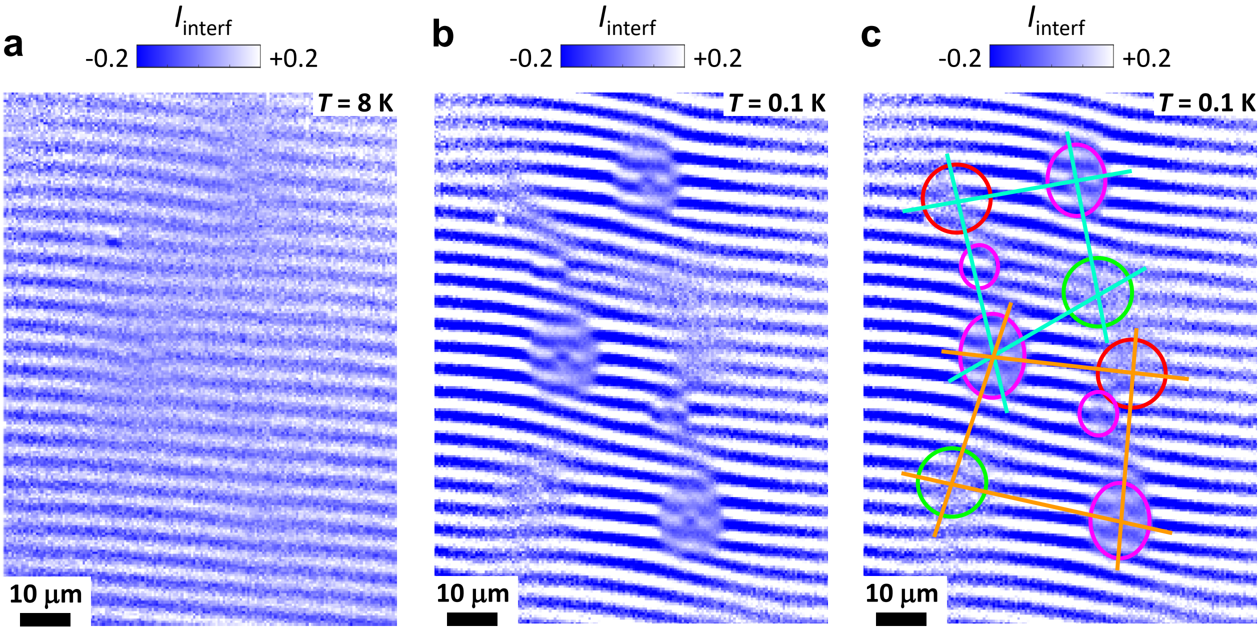

Supplementary Fig. 2. **The features of IX condensate interference patterns.** (a,b) Measured shift-interference patterns $I_{\rm interf}(x, y)$ for IXs in the region of five LBS sources at $T = 8$ K (a) and $T = 0.1$ K (b). (c) Same as (b) with the marked features of IX condensate interference patterns. For a classical exciton gas at high temperatures the interference patterns are featureless (a). For the IX condensate at low temperatures, the interference patterns are complex (b,c). Sharp phase shifts of interference fringes embracing a phase domain of interference fringes in a circular region around each LBS source of IXs are marked by magenta lines (c). These sharp phase shifts and, in turn, the phase domains are associated with the Pancharatnam-Berry phase and are described in Ref. [2]. The right- and left-oriented dislocations in the interference pattern are marked by red and green circles, respectively (c). These interference dislocations originate from the moiré effect in the combined interference patterns of radial IX condensate matter waves propagating from the sources of IXs and are described in this work. All sources participate in the formation of these dislocations. A stronger contribution to the upper (lower) pair of right- and left-oriented dislocations is given by the two upper (lower) strong sources. This is illustrated by cyan (orange) lines between the dislocations and the sources giving stronger contribution. These lines form diamond shapes similar to the diamond shapes formed by the lines connecting interference dislocations and two sources producing them in Supplementary Fig. 1b.

The relation between the second and third features: The position of interference fringes in the source vicinity including the sharp phase shifts of interference fringes around the source allow estimating the IX momenta k as described in Ref. [2]. The ballistic IX propagation described by the multi-source analogue of Eq. (1) in the main text with these values of k produces interference dislocations (Fig. 4b) at the locations, which qualitatively reproduce the experiment (Fig. 4a). IXs propagate over ca. 30 μm from the phase domains in a circular region around each LBS source of IXs to the locations of interference dislocations with the fixed k that shows ballistic IX propagation without scattering over these macroscopic distances.

We note also that no correlation like the one between the sharp phase shifts (second feature) and polarization changes is established between the interference dislocations (third feature) and polarization changes. Some interference dislocations (e.g. the dislocation shown in Fig. 1) are observed in the regions of nearly constant polarization, while for some interference dislocations polarization changes are observed in their vicinity. In the latter case, the Pancharatnam-Berry phase acquired due to the polarization changes may affect the interference fringes, however, for all interference dislocations analyzed here, the polarization-related effects cause no drastic changes in the interference dislocations.

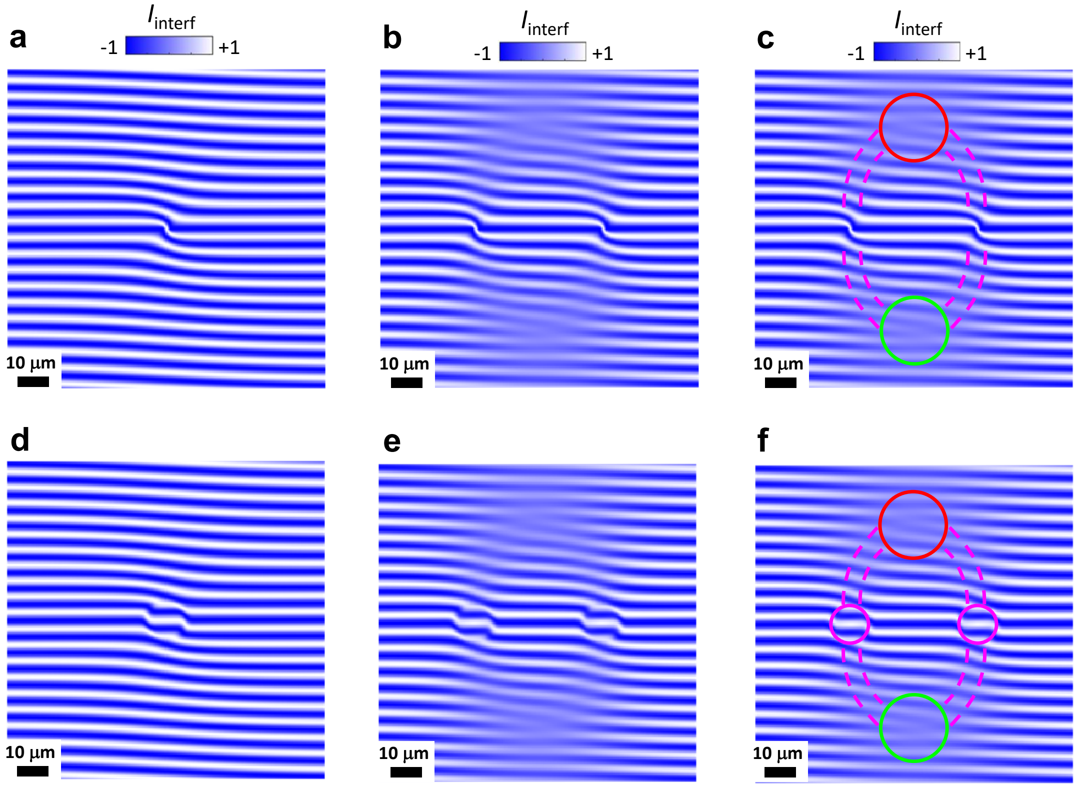

Supplementary Fig. 3. **Simulated exciton interference pattern with interference dislocations and sharp and smooth phase shifts.** (a) Shift-interference pattern $I_{\text{interf}}(x, y)$ for a radial IX condensate matter wave propagating from a source in the center (same as Fig. 3a). (b) $I_{\text{interf}}(x, y)$ for two radial IX condensate matter waves propagating from two sources separated along x (same as Fig. 3b). (c) Same as (b) with the interference dislocations marked by green and red circles and smooth phase shifts of interference fringes marked by dashed magenta lines. (d-f) Same as (a-c) with shown phase domains at the source locations. The phase domains are embraced by sharp phase shifts marked by solid magenta lines. The sharp phase shifts and, in turn, the phase domains in interference patterns are associated with the Pancharatnam-Berry phase and are described in Ref. [2]. The smooth phase shifts marked by dashed magenta lines can be followed from the phase domains in a circular region around each LBS source of IXs to the interference dislocations. Both the interference dislocations and the smooth phase shifts originate from the moiré effect in the combined interference patterns of radial IX condensate matter waves propagating from the sources.

Finally, we discuss the development of understanding of interference dislocations. The presence of interference dislocations (forks) in the IX condensate interference patterns was detected in Ref. [4]. It was understood that phase vortices cannot explain them [4], however the origin of dislocations remained unclear. A model of a ring-shaped source of IXs considered in Ref. [4] was able to produce dislocations, however, these dislocations appeared adjacent to the ring-shaped source and this model did not produce dislocations macroscopic distances away from sources; the separation from sources by macroscopic distances is the key feature of isolated interference dislocations in the experiment.

The isolated interference dislocations observed at macroscopic distances away from the LBS sources originate from the moiré effect in the combined interference patterns of condensate matter waves propagating from different LBS sources. These interference dislocations are described in this work. The other type of interference dislocation, generated by a single LBS source and adjacent to it, is described in Ref. [4] ([21] in the main text). The isolated interference dislocations observed at macroscopic distances away from the LBS sources tend to appear in the regions containing two (or more) strong LBS sources capable to generate the isolated interference dislocations at macroscopic distances away from them as described in this work. The interference dislocations adjacent to the source tend to appear in the regions where an LBS source does not have another strong LBS source in its vicinity.

An interpretation in terms of skyrmions was considered in Ref. [5]. However, as we show in this work, the interference patterns produced by skyrmions (Fig. 2) are drastically different from the experiment (Fig. 1b), indicating

that the observed interference dislocations are not associated with skyrmions.

It was also noted in Ref. [2] that besides the sharp phase shifts in a circular region around each LBS source of IXs (the second feature outlined above), there are smoother phase shifts, which can be followed further up to the interference dislocations, indicating that the dislocations originate from the phase domains. The origin of the dislocations and the relation between the dislocations and the phase domains had been, however, unclear. The origin of the dislocations and the relation between the dislocations and the phase domains are found in this work and outlined in the above paragraphs. Furthermore, the smooth phase shifts, which can be followed from the phase domains in a circular region around each LBS source of IXs to the interference dislocations, are also clarified in this work: The smooth phase shifts appear in the moiré pattern of interference fringes produced by radial IX condensate matter waves propagating from the sources. This is shown in Supplementary Fig. 3 for the case of two IX sources by dashed magenta lines marking the smooth phase shifts. To summarize the development of understanding, this work reports on finding the origin of the isolated interference dislocations separated from the sources by macroscopic distances reaching 30 microns.

Supplementary Note 6: Temperature dependence

Supplementary Fig. 2 shows that the interference dislocations are observed in the IX condensate at low temperatures and disappear in a classical IX gas at high temperatures. In this section, we present the temperature dependence showing how the interference dislocations disappear.

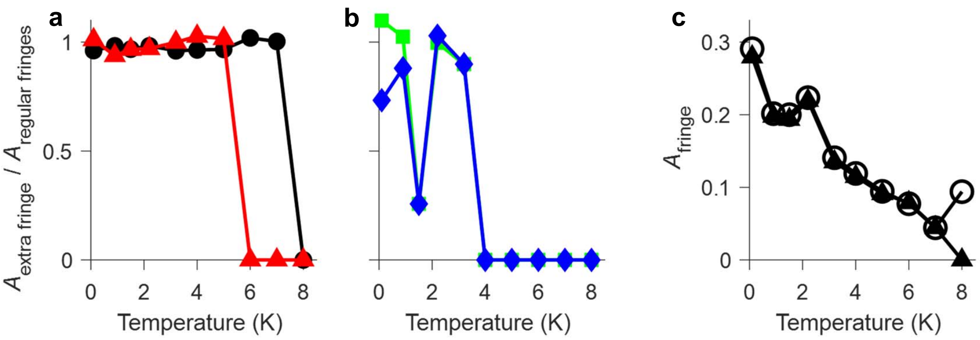

Supplementary Fig. 4. **Temperature dependence.** (a) The relative amplitude of the "extra" interference fringe vs. temperature for the lower left (black points) and right (red triangles) interference dislocations in Supplementary Fig. 2b. The "extra" interference fringes and, in turn, the interference dislocations disappear at ∼ 8 and ∼ 6 K, respectively. (b) The relative amplitude of the "extra" interference fringe vs. temperature for the upper left (green squares) and right (blue diamonds) interference dislocations in Supplementary Fig. 2b. The "extra" interference fringes and, in turn, the interference dislocations disappear at ∼ 4 K. A mutual annihilation of these dislocations is observed at 1.5 K. (c) The amplitudes of "regular" (circles) and "extra" (triangles) interference fringes vs. temperature for the lower left interference dislocation in Supplementary Fig. 2b.

To quantify, the presence of dislocations in the interference pattern, we draw closed contours around the dislocation points. For an interference dislocation, the contour crosses an odd number of interference fringes, so the phase of the interference fringes winds by 2π along the closed contour, indicating a phase singularity in the interference pattern. In contrast, for the case of no dislocation in the interference pattern embraced by the contour, the contour crosses an even number of interference fringes. Therefore, the presence of the interference dislocation can be characterized by the relative amplitude of the "extra" interference fringe along the closed contour.

Supplementary Fig. 4 shows that the disappearance of interference dislocation with increasing temperature is sharp: The relative amplitude of the "extra" fringe stay high up to a certain temperature and then sharply disappears with the further increase of temperature (Supplementary Fig. 4a). Different interference dislocations disappear at different temperatures ranging from ∼ 4 K to ∼ 8 K (Supplementary Fig. 4a,b). The spread of temperatures for the disappearance of interference dislocations may be related to spatial inhomogeneities in the system, the theoretical understanding of this yet need to be developed.

Left- and right- oriented interference dislocations may mutually annihilate. This effect is observed for the upper two dislocations in Supplementary Fig. 2b. These two dislocations approach and suppress each other at 1.5 K (Supplementary Fig. 4b).

The absolute amplitudes of both "regular" interference fringes and the "extra" interference fringe, in the temperature range of its existence, gradually reduce with temperature (Supplementary Fig. 4c). This complies with the gradual reduction of the superfluid density with increasing temperature: A nearly linear reduction of the superfluid density with temperature at temperatures below the Berezinskii-Kosterlitz-Thouless transition is found in the theory [6].

Supplementary Note 7: Shift dependence

The interference dislocations arise from the moiré effect in the combined interference patterns of IX condensate matter waves propagating from multiple sources. These sources are rather frequent, the characteristic distances between them are on the order of few tens of microns (Supplementary Fig. 2b). As outlined in section Methods, the optimal shift in the shift-interferometry experiments should be smaller than the coherence length, smaller than the characteristic sizes of the features in the interference patterns, and larger than the spatial resolution in the experiment. The shift $\delta x = 2$ μm in the experiments outlined above fulfills these requirements.

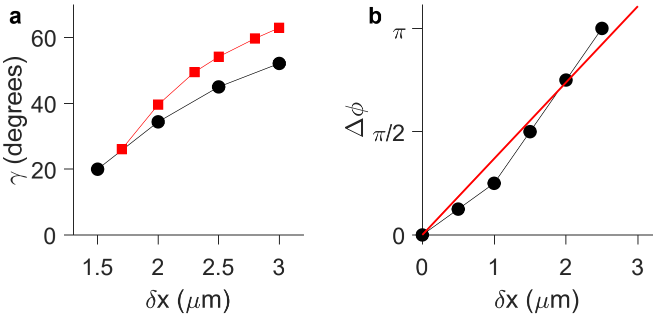

Supplementary Fig. 5. **Shift dependence.** (a) Measured (points) and simulated within the moiré-effect model (red squares) angle γ vs. the shift δx. γ is the angle between the line connecting the sources and the direction from the source to the interference dislocation (Supplementary Fig. 1b). γ determines the location of interference dislocation. The interference dislocation is the lower left interference dislocation in Supplementary Fig. 2b. This interference dislocation is relatively well isolated and is mainly produced by a relatively well isolated pair of lower two strong sources in Supplementary Fig. 2b. The average value is taken for γ for the upper and lower source. Both in the experiment and in the simulation, γ increases with the shift δx. (b) The sharp phase shift of interference fringes embracing the phase domain in the source vicinity vs. the shift δx (points). These sharp phase shifts of interference fringes are proportional to both k and δx [2]. The red line drawn through $\delta x = 2$ μm data corresponds to the IX momentum $k = 1.2$ μm^{-1}.

Following the evolution of interference dislocation with the shift δx can be done with an interference dislocation relatively well isolated from other features of the interference pattern and produced by a relatively well isolated pair of sources. The lower left interference dislocation in Supplementary Fig. 2b is relatively well isolated, it is mainly produced by a relatively well isolated pair of lower two strong sources in Supplementary Fig. 2b. The evolution of this interference dislocation with the shift δx is presented in Supplementary Fig. 5a. The location of interference dislocation is described by angle γ between the line connecting the sources and the direction from the source to the interference dislocation (Supplementary Fig. 1b). In Supplementary Fig. 5a, the experimentally measured values of γ are compared with the values of γ simulated within the moiré-effect model of interference dislocation produced by an isolated pair of sources. Both in the experiment and in the simulation, γ increases with the shift δx (Supplementary Fig. 5a).

An increase of the shift δx also affects the sharp phase shifts of interference fringes embracing the phase domain in the source vicinity. As described in Ref. [2], these sharp phase shifts of interference fringes are proportional to both k and δx. Supplementary Fig. 5b shows that the sharp phase shifts of interference fringes increase with δx. The slope of this increase agrees with the IX momentum $k \sim 1.5$ μm^{-1} estimated from $\delta x = 2$ μm data (Supplementary Fig. 5b).

[1] L.V. Butov, C.W. Lai, D.S. Chemla, Yu.E. Lozovik, K.L. Campman, A.C. Gossard, Observation of Magnetically Induced Effective-Mass Enhancement of Quasi-2D Excitons, *Phys. Rev. Lett.* **87**, 216804 (2001).
[2] J.R. Leonard, A.A. High, A.T. Hammack, M.M. Fogler, L.V. Butov, K.L. Campman, A.C. Gossard, Pancharatnam-Berry phase in condensate of indirect excitons, *Nature Commun.* **9**, 2158 (2018).
[3] A.L. Ivanov, L.E. Smallwood, A.T. Hammack, Sen Yang, L.V. Butov, A.C. Gossard, Origin of the inner ring in photoluminescence patterns of quantum well excitons, *Europhys. Lett.* **73**, 920 (2006).
[4] A.A. High, J.R. Leonard, A.T. Hammack, M.M. Fogler, L.V. Butov, A.V. Kavokin, K.L. Campman, A.C. Gossard, Spontaneous coherence in a cold exciton gas, *Nature* **483**, 584 (2012).
[5] D.V. Vishnevsky, H. Flayac, A.V. Nalitov, D.D. Solnyshkov, N.A. Gippius, G. Malpuech, Skyrmion Formation and Optical Spin-Hall Effect in an Expanding Coherent Cloud of Indirect Excitons, *Phys. Rev. Lett.* **110**, 246404 (2013).
[6] Daniel S. Fisher, P.C. Hohenberg, Dilute Bose gas in two dimensions, *Phys. Rev. B* **37**, 4936 (1988).
