# Supplementary material for: Moiré pattern of interference dislocations in condensate of indirect excitons
Source: Nat Commun. 2021 Feb 19;12:1175. doi: 10.1038/s41467-021-21353-7 (PMC7895953; doi:10.1038/s41467-021-21353-7)
Supplement: Supplementary file 2 — Description of Additional Supplementary Files [file 41467_2021_21353_MOESM2_ESM.docx]

File Name: Supplementary Movie 1

Description: A movie showing how the interference dislocations appear and how their locations change when the two combining patterns of interference fringes move relative to each other. In this movie, the bottom pattern of interference fringes is given by the first term in Eq. (1) in the main text and the top pattern of interference fringes is given by the second term in Eq. (1) in the main text. The top pattern is semitransparent so that the intensities of the bottom and top patterns add with the same weight in the overlapping region, visualizing Eq. (1) in the main text and showing the moiré pattern of interference dislocations. The overlapping region is marked by a dashed contour.

File Name: Supplementary Movie 2

Description: Same as Supplementary Movie 1 with the interference dislocations marked by green and red circles.
