# Supplementary figures and images for: Moiré pattern of interference dislocations in condensate of indirect excitons
Source: Nat Commun. 2021 Feb 19;12:1175. doi: 10.1038/s41467-021-21353-7 (PMC7895953; doi:10.1038/s41467-021-21353-7)

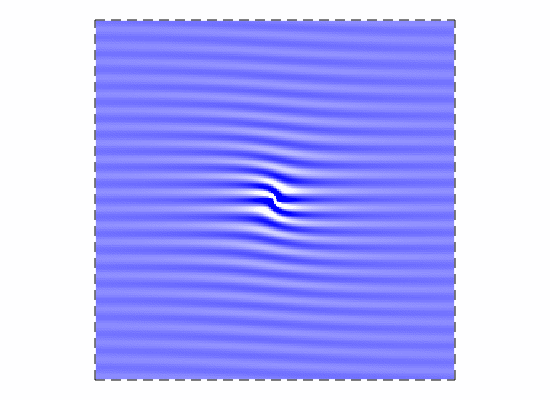

Supplement: Supplementary file 3 — Supplementary Movie 1 [file 41467_2021_21353_MOESM3_ESM.gif]

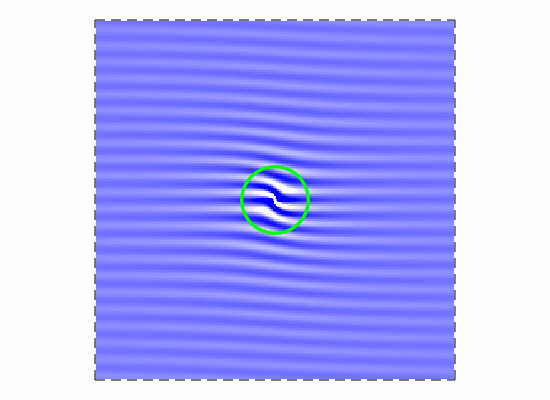

Supplement: Supplementary file 4 — Supplementary Movie 2 [file 41467_2021_21353_MOESM4_ESM.gif]
